# Supplementary material for: Evaluating the impact of a rapid response system on survival of patients with cancer undergoing emergency surgery for acute abdomen: A single-center retrospective cohort study
Source: PLoS One. 2026 Jan 30;21(1):e0341616. doi: 10.1371/journal.pone.0341616 (PMC12857990; doi:10.1371/journal.pone.0341616)
Supplement: S1 Table — This table presents demographic factors, cancer-related characteristics, illness severity markers, perioperative time intervals, and postoperative outcomes in patients undergoing emergency surgery for acute abdomen. Variables compared include age, sex, cancer type and stage, APACHE II and SOFA scores, lactate level, organ support use, and survival to discharge. Differences between the groups were assessed using appropriate statistical tests, with results reported as mean (SD), median (IQR), or n (%). (DOCX) [file pone.0341616.s001.docx]

**S1 Table. Comparison of clinical characteristics between patients admitted during hours with Rapid Response System coverage and those admitted outside coverage hours.**

|  | **RRS Coverage hours**  **(N=274) (%)** | **Outside coverage hours**  **(N=62) (%)** | **Total**  **(N=336) (%)** | **P value** |
| --- | --- | --- | --- | --- |
| Age (years) | 69.00 (60.00–78.00) | 69.00 (63.00–77.00) | 69.00 (60.00–77.00) | 0.656 |
| Sex, female | 124 (45.42) | 31 (50.00) | 155 (46.27) | 0.609 |
| Type of Cancer |  |  |  | 0.624 |
| Others | 24 (8.76) | 10 (16.13) | 34 (10.12) |  |
| Gynecologic | 63 (22.99) | 13 (20.97) | 76 (22.62) |  |
| Hematology | 8 (2.92) | 1 (1.61) | 9 (2.68) |  |
| Hepatobiliary | 28 (10.22) | 8 (12.90) | 36 (10.71) |  |
| Lower gastrointestinal | 63 (22.99) | 12 (19.35) | 75 (22.32) |  |
| Lung | 30 (10.95) | 8 (12.90) | 38 (11.31) |  |
| Upper gastrointestinal | 58 (21.17) | 10 (16.13) | 68 (20.24) |  |
| Stage |  |  |  | 0.550 |
| Localized | 130 (56.03) | 38 (61.29) | 168 (57.14) |  |
| Metastatic | 102 (43.97) | 24 (38.71) | 126 (42.86) |  |
| Site of Operation |  |  |  | 0.009 |
| Others | 4 (1.46) | 6 (9.68) | 10 (2.98) |  |
| Hepatobiliary | 18 (6.57) | 2 (3.23) | 20 (5.95) |  |
| Lower gastrointestinal | 105 (38.32) | 19 (30.65) | 124 (36.90) |  |
| Lung | 13 (4.74) | 6 (9.68) | 19 (5.65) |  |
| Upper gastrointestinal | 134 (48.91) | 29 (46.77) | 163 (48.51) |  |
| Cause of Operation |  |  |  | 0.768 |
| Others | 12 (4.38) | 4 (6.45) | 16 (4.76) |  |
| Bleeding | 39 (14.23) | 7 (11.29) | 46 (13.69) |  |
| Ischemia | 28 (10.22) | 6 (9.68) | 34 (10.12) |  |
| Obstruction | 34 (12.41) | 5 (8.06) | 39 (11.61) |  |
| Perforation | 161 (58.76) | 40 (64.52) | 201 (59.82) |  |
| ASA |  |  |  | 0.314 |
| Low (<4) | 216 (78.83) | 53 (85.48) | 269 (80.06) |  |
| High (≥4) | 58 (21.17) | 9 (14.52) | 67 (19.94) |  |
| Hypertension | 102 (37.36) | 25 (40.32) | 127 (37.91) | 0.773 |
| Diabetes Mellitus | 62 (22.71) | 19 (30.65) | 81 (24.18) | 0.249 |
| Cardiovascular disease | 9 (3.30) | 4 (6.45) | 13 (3.88) | 0.271 |
| Chronic obstructive pulmonary disease | 12 (4.38) | 1 (1.61) | 13 (3.87) | 0.476 |
| Chronic kidney disease | 4 (1.46) | 2 (3.23) | 6 (1.79) | 0.306 |
| Liver disease | 7 (2.55) | 2 (3.23) | 9 (2.68) | 0.674 |
| APACHE II score | 25.00 (16.00–31.00) | 28.00 (23.00–32.00) | 26.00 (19.00–31.00) | 0.002 |
| SOFA score | 4.00 (2.00–7.00) | 4.50 (2.00–7.00) | 4.00 (2.00–7.00) | 0.940 |
| Lactic Acid (mg/dL) | 30.55 (15.00–54.80) | 34.90 (18.20–61.20) | 32.15 (15.95–58.10) | 0.335 |
| Preoperative Fluid administration (L) | 0.30 (0.30–1.00) | 0.55 (0.30–1.10) | 0.30 (0.30–1.00) | 0.224 |
| ICU Admission |  |  |  | < 0.001 |
| Postoperative | 119 (43.43) | 43 (69.35) | 162 (48.21) |  |
| Preoperative | 155 (56.57) | 19 (30.65) | 174 (51.79) |  |
| Diagnosis to ICU admission (hour) | 7.12 (4.15–12.05) | 9.15 (7.00–10.43) | 7.72 (4.42–11.58) | 0.114 |
| Diagnosis to Operation (hour) | 5.20 (2.63–10.05) | 7.66 (6.20–10.97) | 5.95 (3.16–10.14) | < 0.001 |
| Diagnosis to Antibiotics administration (hour) | 2.23 (1.12–5.00) | 3.12 (1.78–4.88) | 2.43 (1.18–4.98) | 0.116 |
| Vasopressor |  |  |  |  |
| Preoperative | 79 (28.83) | 15 (24.19) | 94 (27.98) | 0.563 |
| Postoperative | 149 (54.38) | 40 (64.52) | 189 (56.25) | 0.190 |
| Duration (days) | 1.53 (0.67–2.58) | 1.14 (0.56–3.55) | 1.48 (0.60–2.64) | 0.788 |
| Mechanical ventilation |  |  |  |  |
| Preoperative | 37 (13.50) | 12 (19.35) | 49 (14.58) | 0.327 |
| Postoperative | 147 (53.65) | 34 (54.84) | 181 (53.87) | 0.977 |
| Duration (days) | 2.00 (2.00–4.00) | 4.50 (2.00–13.00) | 3.00 (2.00–5.00) | 0.003 |
| CRRT | 12 (4.38) | 6 (9.68) | 18 (5.36) | 0.115 |
| ICU length of stay (days) | 1.91 (0.81–4.61) | 2.60 (0.67–5.61) | 1.98 (0.78–4.79) | 0.788 |
| Postoperative hospital stay (days) | 20.64 (11.82–37.12) | 20.70 (11.98–38.02) | 20.64 (11.82–37.52) | 0.507 |
| Survival discharge |  |  |  | 0.018 |
| Death | 56 (20.44) | 22 (35.48) | 78 (23.21) |  |
| Survival | 218 (79.56) | 40 (64.52) | 258 (76.79) |  |

Values are presented as mean (SD), median (IQR), or n (%).

APACHE II, Acute Physiology and Chronic Health Evaluation II; ASA, American Society of Anesthesiologists; CRRT, continuous renal replacement therapy; ICU, Intensive Care Unit; RRS, rapid response system; SOFA, Sequential Organ Failure Assessment.
