# Supplementary material for: Evaluating the impact of a rapid response system on survival of patients with cancer undergoing emergency surgery for acute abdomen: A single-center retrospective cohort study
Source: PLoS One. 2026 Jan 30;21(1):e0341616. doi: 10.1371/journal.pone.0341616 (PMC12857990; doi:10.1371/journal.pone.0341616)
Supplement: S2 Table — Multivariable logistic regression was performed including RRS implementation, ICU admission timing, metastatic disease, APACHE II score, lactic acid level, CRRT use, and study year. The interaction term (RRS × preoperative ICU admission) was not statistically significant, indicating no evidence of a differential effect of RRS on survival based on timing of ICU admission. Adjusted odds ratios (ORs) with 95% confidence intervals (CIs) are presented. APACHE II, Acute Physiology and Chronic Health Evaluation II; CRRT, continuous renal replacement therapy; ICU, Intensive Care Unit; RRS, rapid response system. (DOCX) [file pone.0341616.s002.docx]

**S2 Table. Interaction Analysis of RRS Implementation and Preoperative ICU Admission on Survival**

|  | **Multivariable analysis** | |
| --- | --- | --- |
|  | **Adjusted OR (95% CI)** | **P-value** |
| RRS | 1.642 (0.189–11.67) | 0.620 |
| ICU admission |  |  |
| Postoperative | Reference |  |
| Preoperative | 0.101 (0.011–0.623) | 0.022 |
| Metastatic | 0.174 (0.053–0.488) | 0.002 |
| APACHE II | 0.924 (0.861–0.986) | 0.021 |
| Lactic acid (mg/dL) | 1.001 (0.985–1.017) | 0.942 |
| CRRT | 0.186 (0.030–0.947) | 0.051 |
| Study year  Interaction: RRS × Preoperative ICU admission | 1.239 (0.909–1.717)  3.055 (0.299–37.30) | 0.183  0.352 |

APACHE II, Acute Physiology and Chronic Health Evaluation II; CRRT, continuous renal replacement therapy; ICU, Intensive Care Unit; RRS, rapid response system.
