# Supplementary material for: Evaluating the impact of a rapid response system on survival of patients with cancer undergoing emergency surgery for acute abdomen: A single-center retrospective cohort study
Source: PLoS One. 2026 Jan 30;21(1):e0341616. doi: 10.1371/journal.pone.0341616 (PMC12857990; doi:10.1371/journal.pone.0341616)
Supplement: S3 Table — Observed survival reflects the actual survival rate among patients who deteriorated outside coverage hours in the pre-RRS period. Counterfactual survival represents the predicted probability of survival if these patients had access to RRS activation, based on an adjusted logistic regression model incorporating RRS implementation, coverage status, APACHE II score, SOFA score, lactate level, age, and cancer stage. The absolute difference demonstrates the estimated magnitude of survival improvement under hypothetical RRS availability during these hours. (DOCX) [file pone.0341616.s003.docx]

**S3 Table. Observed and Counterfactual Survival During Outside Coverage Hours**

| Variable | **Survival Probability (%)** | **Description** |
| --- | --- | --- |
| Observed survival during outside coverage hours (Pre-RRS) | 59.4% | Actual survival among patients evaluated outside RRS coverage hours before RRS implementation |
| Counterfactual survival with RRS assumed during outside coverage hours | 71.0% | Predicted survival if patients outside coverage hours had access to RRS activation (adjusted model) |
| Absolute difference | + 11.6 percentage points | Estimated survival improvement attributable to hypothetical RRS coverage during these hours |

Counterfactual predictions were generated using an adjusted logistic regression model including RRS implementation, coverage status, APACHE II score, SOFA score, lactate, age, and cancer stage.
