# Supplementary figures and images for: Evaluating the impact of a rapid response system on survival of patients with cancer undergoing emergency surgery for acute abdomen: A single-center retrospective cohort study
Source: PLoS One. 2026 Jan 30;21(1):e0341616. doi: 10.1371/journal.pone.0341616 (PMC12857990; doi:10.1371/journal.pone.0341616)

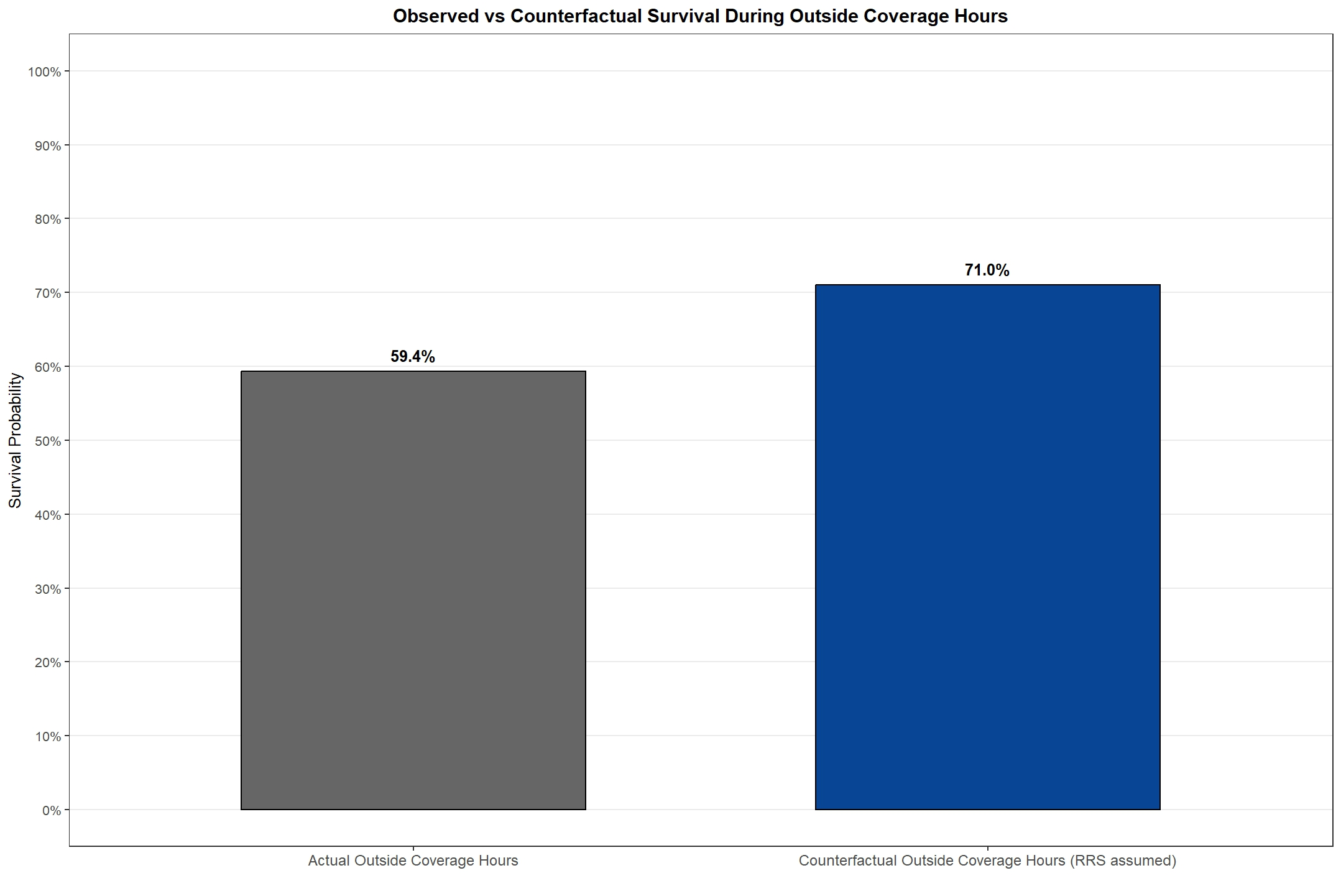

Supplement: S1 Fig — The left bar shows the observed survival probability (59.4%) among patients who deteriorated outside RRS coverage hours. The right bar displays the counterfactual survival probability (71.0%) predicted under a hypothetical scenario in which full RRS activation was available during these hours. Counterfactual estimates were generated using an adjusted logistic regression model including RRS implementation, coverage status, APACHE II score, SOFA score, lactate level, age, and cancer stage. The difference illustrates the potential survival benefit associated with extending RRS availability to periods currently without operational coverage. (TIF) [file pone.0341616.s004.tif]
