# Supplementary material for: Evaluating the impact of a rapid response system on survival of patients with cancer undergoing emergency surgery for acute abdomen: A single-center retrospective cohort study
Source: PLoS One. 2026 Jan 30;21(1):e0341616. doi: 10.1371/journal.pone.0341616 (PMC12857990; doi:10.1371/journal.pone.0341616)
Supplement: Attachment — Submitted filename: Response to reviewer 2.docx [file pone.0341616.s007.docx]

**Response to Reviewer’s Comments**

We sincerely appreciate the reviewer’s thoughtful and constructive comments. Your feedback greatly strengthened the clarity and rigor of our manuscript. We carefully revised the relevant sections in response to your suggestions, and a detailed point-by-point explanation is provided below along with the corresponding revised text from the manuscript.

Question 1

It is verified that there is no logical contradiction between the two pieces of information in the manuscript PONED2516230_reviewer.pdf, but the temporal relationship needs to be clarified.

The study included 274 patients who underwent emergency surgery for acute abdomen between March 2016 and August 2023; this time period represents the patient enrollment and data collection phase of the retrospective cohort study, which is consistent with the retrospective nature of the study.

The data access period for the manuscript is specified as October 14, 2024, to December 14, 2024, which corresponds to the poststudy data verification and manuscript preparation phase. Since the study was approved by the Institutional Review Board (IRB) of the National Cancer Center with approval number NCC 20240268, the data access period is set after IRB approval to ensure compliance with ethical requirements for retrospective data use.

Additionally, the manuscript correctly states that "the requirement for informed consent from patients was waived owing to the retrospective nature of this study," which aligns with common ethical practices for retrospective cohort studies (i.e., waiving informed consent when historical data are used and patient privacy is protected).

: We thank the reviewer for this helpful comment. There is no contradiction between the study period and the data access period; however, we agree that clarifying the temporal relationship improves readability. Patient enrollment and clinical data generation occurred between March 2016 and August 2023, while access to the electronic medical records for data verification took place from October 14 to December 14, 2024, after obtaining IRB approval (NCC 2024-0268). We revised the Ethics Statement accordingly.

The Revised text added to the manuscript (Material and method section, Page 4-5):

*“This study was conducted in accordance with the principles of the Declaration of Helsinki and was approved by the Institutional Review Board of the National Cancer Center, Korea (approval no.: NCC 2024-0268). Patients who underwent emergency surgery for acute abdomen between March 2016 and August 2023 were included in this retrospective cohort. Access to the electronic medical records for data verification was from October 14, 2024 to December 14, 2024, following IRB approval. The requirement for informed consent was waived because the study used previously recorded data without direct patient contact.”*

Question 2: Sentence Structure Errors

1. Tense Inconsistency:

Original sentence (Ethics Statement section): "This study was conducted in accordance with... and was approved... The data access period for the manuscript is from October 14, 2024... to December 14, 2024"

Issue: The first half uses the past tense ("was conducted," "was approved") to describe completed study procedures, while the second half incorrectly uses the present tense ("is") for the data access period. Since the data access period is a prespecified window approved by the IRB (and the manuscript is in the submission stage), the past tense should be used to maintain consistency.

Revised sentence: "This study was conducted in accordance with... and was approved... The data access period for the manuscript was from October 14, 2024... to December 14, 2024".

: We agree that the use of the present tense created inconsistency in describing the data access period. The sentence was revised to the past tense to accurately reflect the IRB-approved timeframe for data verification. This issue has now been fully corrected as part of the revised Ethics Statement.

The Revised text added to the manuscript (Material and method section, Page 4-5):

*“Access to the electronic medical records for data verification was from October 14, 2024 to December 14, 2024, following IRB approval.”*

2. Missing Logical Conjunctions:

Original sentences (Abstract section): "Kaplan–Meier analysis confirmed better survival over time in the postRRS group (logrank p = 0.039). Time intervals from diagnosis to critical interventions... were significantly shorter following RRS implementation"

Issue: The two sentences describe complementary findings of the study (improved survival and shortened intervention time), but lack a conjunction to clarify their parallel or causal relationship, leading to disjointed logic.

Revised sentences: "Kaplan–Meier analysis confirmed better survival over time in the postRRS group (logrank p = 0.039). Meanwhile, time intervals from diagnosis to critical interventions... were significantly shorter following RRS implementation"

(The conjunction "Meanwhile" emphasizes the parallelism between the two key results, strengthening logical coherence).

: Thank you for pointing out the need for clearer logical connection in the Abstract. During the revision process, we substantially updated the Abstract to reflect the expanded analyses (including postoperative SOFA trajectories and the counterfactual survival model). As a result, the specific sentences noted in the reviewer’s comment no longer appear in the revised version. We ensured that transitions between key findings—survival improvement, timeliness of intervention, and attenuation of organ dysfunction—are now clearly articulated using appropriate conjunctions and sequential phrasing. If further adjustment to the flow of the Abstract is recommended, we would be happy to revise it again.

Question 3: Insufficient Innovation

1. Limitations of Study Design Restricting Innovative Generalization:

The study adopts a single center retrospective cohort design. Although the sample size (274 patients) was shown to have sufficient statistical power via posthoc analysis (power = 1.0 for Cohen’s d = 0.8), single center data cannot rule out confounding effects from "institution specific clinical protocols" (e.g., concurrent improvements in anesthetic techniques or postoperative monitoring protocols). This limits the external validity of the innovative conclusions.

Recommendation:

Supplement a "comparative analysis of baseline characteristics between cases during RRS operational hours and nonoperational hours" to confirm that excluded cases (11:00 PM–6:00 AM) do not introduce selection bias.

Propose a "multicenter data validation plan" (e.g., collaborating with other tertiary cancer centers to replicate the study) to verify whether RRS exerts consistent effects across different institutions, thereby enhancing the generalizability of the innovative findings.

: A comparative analysis between patients presenting during RRS coverage hours and those presenting outside coverage hours has been added to address the possibility of selection bias. Baseline demographic and oncologic factors were largely comparable between groups, while differences were mainly observed in clinical severity and time-to-intervention metrics. These results have been incorporated into the Results section and are detailed in Supplementary Table S1. In addition, the Limitations section has been expanded to explicitly acknowledge the influence of institution-specific clinical protocols and to state the necessity of multicenter validation. The revised text notes that replication across tertiary cancer centers is required to confirm whether the observed RRS effects are consistent in different hospital systems.

The Revised text added to the manuscript (Result section, Page 9-10):

*“To assess whether excluding patients who presented outside RRS coverage hours introduced systematic differences, we compared patients who deteriorated during RRS coverage hours (N=274) with those who presented outside coverage hours (N=62) ... Other perioperative parameters and postoperative outcomes showed no significant differences (Supplementary Table S1).”*

Limitation section (Page 19):

*“Future studies with larger cohorts across multiple tertiary cancer centers will be important to verify whether the observed effects are consistent in different clinical environments. Evaluating continuous, 24-hour RRS operation and integrating standardized, intervention-level documentation will help clarify which components most contribute to improved prognosis.”*

2. Insufficient Depth of Innovation:

The study does not explore the "specific effective components of RRS"—for example, which subcomponents of the RRS team (e.g., "intensivistled bedside assessment," "realtime vital sign monitoring by nurses," or "multidisciplinary rapid consultation") contribute more to improved prognosis. Without stratified or subgroup analyses to decompose these components, the innovative conclusion remains at the macro level of "RRS is effective," lacking detailed guidance for clinical practice.

Recommendation:

Add "subgroup analyses of the association between different RRS interventions (e.g., bedside stabilization only, ICU transfer, or emergency surgery coordination) and prognosis" to identify the most impactful components of RRS. This will elevate the clinical guiding value of the innovative findings by providing actionable insights for optimizing RRS implementation.

: We appreciate the reviewer’s insightful comment regarding the need to identify which specific components of the RRS most strongly contribute to improved outcomes. We fully agree that decomposing individual RRS interventions would provide more actionable guidance for clinical practice. However, in our institution, the RRS is implemented as an integrated bundle, and individual components—such as bedside assessment, stabilization procedures, ICU transfer coordination, or expedited surgical preparation—are not recorded as discrete variables within the electronic medical record. Because each activation involves a combination of these actions, retrospective separation and classification of specific subcomponents was not feasible without introducing significant misclassification bias. Nevertheless, to acknowledge this important point, we have revised the Limitations section to emphasize that the current study design does not allow identification of the most effective components of the RRS. We have also highlighted that future prospective or multicenter studies with structured documentation of RRS activities will be essential to determine which elements contribute most to improved outcomes.

The Revised text added to the manuscript (Limitation section, Page 19):

*“Finally, although this study demonstrated improved outcomes following RRS implementation, detailed information on the specific types of RRS interventions was not consistently available, making it difficult to determine which elements of the RRS contributed most to the observed effect. Future studies with larger cohorts across multiple tertiary cancer centers will be important to verify whether the observed effects are consistent in different clinical environments. Evaluating continuous, 24-hour RRS operation and integrating standardized, intervention-level documentation will help clarify which components most contribute to improved prognosis.”*

Question 4: Incomplete Logical Closure in Study Design

Issue 1: Uncontrolled Confounding from RRS Operational Hours

The manuscript explicitly states that RRS operates only "on weekdays from 6:00 AM to 11:00 PM" and excludes cases occurring between "11:00 PM and 6:00 AM." However, it fails to:

Analyze "baseline differences (e.g., disease severity, intervention measures) between excluded cases (nonoperational hours) and included cases (operational hours)";

Explain whether "cases during RRS operational hours included patients whose condition deteriorated at night but were delayed until daytime for RRS activation."

These omissions may introduce "mild disease bias" in the included sample (e.g., excluding more severely ill patients who deteriorate at night), weakening the causal inference that "RRS improves prognosis" .

Issue 2: Inadequate Explanation of Baseline Imbalance

Results show that the postRRS group had:

A higher proportion of metastatic disease (56.03% vs. 31.90%, p < 0.001);

Higher APACHE II scores (27 vs. 23, p = 0.009);

yet a significantly higher survival rate (85.27% vs. 74.48%, p = 0.039). The authors only attribute this to "timely intervention by RRS" but fail to rule out confounding factors from "concurrent improvements in hospitalwide clinical practices" (e.g., advances in emergency surgical techniques, optimized postoperative infection control, or enhanced ICU monitoring) between 2016–2019 (preRRS) and 2019–2023 (postRRS), leading to an incomplete causal chain .

Recommendations

1. Supplement a "table comparing baseline characteristics of cases during RRS operational vs. nonoperational hours" to justify the rationality of excluding nonoperational hour cases (e.g., confirming no significant differences in disease severity between the two groups).

: We agree with the reviewer’s concern regarding potential bias introduced by excluding cases during nonoperational RRS hours. To address this, we conducted an additional comparison of patients presenting during coverage versus outside coverage hours and provided the results in Supplementary Table S1, with corresponding descriptions added to the Results section. Furthermore, to evaluate the potential clinical impact of limited RRS availability, we performed an exploratory counterfactual analysis, now presented in Supplementary Table S3 and Figure S1.

The Revised text added to the manuscript (Statistical analysis section, Page 5-6):

*“To assess whether excluding cases presenting outside RRS coverage hours introduced systematic bias, patients evaluated during operational and nonoperational periods were compared separately, with results summarized in Supplementary Table S1.” And “In a supplementary counterfactual analysis, an adjusted logistic regression model … Counterfactual survival probabilities were … This exploratory analysis was conducted to quantify the potential benefit of extending RRS operation to nighttime hours and does not replace the primary regression findings (Table S3 and Figure S1).”*

Result section (Page 9-10):

*“To assess whether excluding patients who presented outside RRS coverage hours … Baseline demographic and oncologic characteristics, including age, sex, cancer type, disease stage, and most comorbidities, were broadly comparable between groups. … parameters and postoperative outcomes showed no significant differences (Supplementary Table S1).*

*To further assess the potential clinical impact of limited RRS … This reflects an absolute survival difference of 11.6 percentage points. Detailed estimates are provided in Supplementary Table S3, and a graphical summary is shown in Supplementary Figure S1.”*

2. Use "propensity score matching (PSM)" to balance baseline variables (e.g., metastatic disease status, APACHE II scores) between the preRRS and postRRS groups; alternatively, include "study year" as a confounding variable in the multivariable regression model to explicitly rule out the impact of concurrent improvements in medical standards.
 : We recognize the reviewer’s concern regarding potential confounding arising from temporal improvements in clinical practice. Although propensity score matching (PSM) was not applied because this study used a pre–post design rather than parallel cohorts, we addressed this issue by explicitly adjusting for study year as a covariate in the multivariable logistic regression model. This approach allows the analysis to account for secular trends and institutional improvements independent of RRS implementation. The revised Statistical Analysis and Result sections now clarify this rationale and describe the inclusion of study year as a confounder.

The Revised text added to the manuscript (Statistical analysis section, Page 5):

*“To account for secular changes in clinical practice over the study period, study year was incorporated as a covariate.”*

Result section (Page 13-14):

*“Univariable logistic regression identified … Organ dysfunction category and study year were evaluated but did not demonstrate significant associations with survival.*

*In the multivariable analysis…Lactate level and organ dysfunction category were not retained in the final model, and study year was evaluated as a covariate to account for temporal improvement in institutional practice but was not independently associated with survival (Table 3).”*

Question 5: Unsubstantiated Variable Selection Criteria

The manuscript mentions that "variables with p < 0.2 in univariable analysis were included in the multivariable regression" but provides no justification for this criterion (e.g., whether it is based on prior studies in the field or preanalysis validation). Additionally, it fails to report the reasons for excluding variables such as "SOFA score and lactate level" from the multivariable model. This may lead to the omission of key confounders, undermining the reliability of the results .

Recommendation:

In the "Statistical Analysis" section, supplement:

1. Literature support for the p < 0.2 criterion (e.g., citing methodological studies that recommend this threshold for variable selection in observational studies);

2. Preanalysis results (e.g., correlation analysis between variables) to explain why variables like SOFA score and lactate level were excluded (e.g., high collinearity with APACHE II score) .

: We clarified these points in the revised Statistical Analysis section. A methodological reference that supports the use of a p < 0.2 threshold for variable selection in observational studies was added (now cited as Reference 20: Vittinghoff et al., Regression Methods in Biostatistics). This addition required renumbering of subsequent references. We also explicitly stated the rationale for variable selection among APACHE II, SOFA score, and lactate. As described in the revised section, APACHE II and SOFA share overlapping physiologic domains; therefore, APACHE II was retained as the primary composite severity indicator, while lactate was included as an independent biochemical marker. This reflects the actual analytic approach used in the study. The corresponding description has been incorporated into the Statistical Analysis section.

The Revised text added to the manuscript (Statistical analysis section, Page 5):

*“Univariable analyses were performed initially, and variables with p values below 0.2 were entered into the multivariable model according to established methodological recommendations (20). … Because physiologic severity could be redundantly represented by multiple variables, we examined the relationship between APACHE II, SOFA score, and lactate. Although variance inflation factors did not indicate harmful multicollinearity, APACHE II and SOFA score capture overlapping physiologic domains; therefore, APACHE II was retained as the primary composite severity indicator, while lactate was included as an independent biochemical marker.”*

Question 6: Lack of Interaction Analysis

The results show that "preoperative ICU admission was significantly associated with poor prognosis (adjusted OR = 0.154, p < 0.001)." However, the study does not analyze "whether RRS improves the prognosis of patients with preoperative ICU admission" (i.e., the interaction between RRS and preoperative ICU admission). This gap prevents clarification of RRS’s intervention value for the "most severely ill subgroup," limiting the depth of logical analysis .

Recommendation:

1. Add an interaction term "RRS × preoperative ICU admission" to the multivariable logistic regression model;

2. Report the adjusted OR and pvalue of the interaction term to determine whether RRS exerts differential effects on patients with preoperative vs. postoperative ICU admission. For example, if the interaction term is significant, it could indicate that RRS is more effective for patients with preoperative ICU admission (a severely ill subgroup) .

: Following this comment, we added an interaction term (RRS × preoperative ICU admission) to the multivariable logistic regression model. The interaction was not statistically significant, indicating that the effect of RRS did not differ between patients with preoperative and postoperative ICU admission. These results were added to the manuscript as Supplementary Table S2, and corresponding descriptions were incorporated into the Statistical analysis, Results and Discussion sections.

The Revised text added to the manuscript (Statistical analysis section, Page 5):

*“To assess whether excluding cases presenting outside RRS coverage hours introduced systematic bias, patients evaluated during operational and nonoperational periods were compared separately, with results summarized in Supplementary Table S1. Finally, to explore whether the effect of the RRS differed in the most critically ill subgroup, an interaction term between RRS implementation and preoperative ICU admission was incorporated into the multivariable model, and full model results are presented in Supplementary Table S2.”*

Result section (Page 13):

*“An interaction analysis was additionally performed to assess whether the effect of RRS implementation differed between patients requiring preoperative versus postoperative ICU admission. The interaction term was not statistically significant, indicating that RRS did not demonstrate a differential impact based on timing of ICU admission (Supplementary Table S2).”*

Discussion section (Page 18):

*“These findings are consistent with previous reports showing that early severe physiological derangement is strongly associated with adverse outcomes in high-risk surgical populations [26–27]. In the subgroup interaction analysis, RRS implementation did not show a differential survival effect between preoperative and postoperative ICU admissions, suggesting that the beneficial impact of RRS was consistent across both levels of preoperative stability.”*

Limitation section (Page 19):

*“Third, the use of convenience sampling may have introduced selection bias, … Additionally, although we examined the interaction between RRS implementation and preoperative ICU admission, the study was not powered to detect subgroup-specific effects, and these results should be interpreted cautiously. Furthermore…”*

Question 7: Incomplete "InterventionPrognosis" Causal Chain

The manuscript claims that "RRS shortens key intervention time windows → improves survival" but fails to explain why shortened time windows lead to better prognosis. For example:

Whether shorter antibiotic administration time reduces sepsis incidence;

Whether shorter ICU admission time decreases the risk of multiple organ failure.

The lack of "intermediate outcome measures" (e.g., sepsis, organ failure) creates a break in the causal chain between "intervention measures" and "prognosis improvement" .

Recommendation:

Supplement "group comparisons of intermediate outcome measures," such as:

Incidence of sepsis (30day postsurgery);

Incidence of multiple organ failure (within 7 days of ICU admission);

This will complete the causal chain: "RRS → shortened time windows → reduced complications → improved survival," strengthening the logical rigor of the conclusion .

: We agree that clarifying the intermediate link between shortened intervention times and improved survival enhances the causal coherence of the study. In our dataset, postoperative 30-day sepsis incidence could not be reliably ascertained; therefore, in accordance with the methods prespecified in the revised manuscript, we used serial SOFA score trajectories within 7 days after surgery as the validated indicator of organ dysfunction progression. As described in the Materials and Methods section, organ failure trajectory was assessed using the established Sepsis-3 threshold (ΔSOFA ≤ –2: improvement; –1 to +1: stability; ≥2: progression). Based on this framework, we re-evaluated postoperative organ dysfunction and supplemented these findings with additional comparisons of vasopressor and ventilator requirements at postoperative day 7. These analyses demonstrated that RRS implementation was associated with shorter times to ICU admission, surgery, and antibiotic administration, which corresponded with lower SOFA scores on postoperative day 7, more favorable SOFA trajectories, and reduced need for organ support. These intermediate improvements provide mechanistic support for the observed survival benefit in the post-RRS group. The corresponding revisions have been added to the Materials and Methods, Results, and Discussion sections of the revised manuscript.

The Revised text added to the manuscript (Material and method section, Page 4):

*“Organ failure trajectory was assessed using changes in SOFA score from ICU admission to day 7. Because a difference of at least 2 points in the SOFA score represents clinically meaningful organ dysfunction in the Sepsis-3 definition, trajectories were categorized as improvement (ΔSOFA ≤ –2), stability (ΔSOFA –1 to +1), or progression (ΔSOFA ≥ 2). This approach extends established methods of serial SOFA evaluation by applying a validated threshold to distinguish meaningful changes in organ failure status during the early postoperative course (19).”*

Result section (Page 6-7)

*“Mechanical ventilation was required in 13.50% of patients preoperatively and 53.65% postoperatively, with a median duration of 2 days. On postoperative day 7, vasopressor use was observed in 5.95% of patients and mechanical ventilation in 9.92%. The median SOFA score on postoperative day 7 was 3, and the median ΔSOFA was −1. Organ dysfunction patterns were categorized as improvement (43.15%), stability (35.08%), or progression (21.77%) based on these serial SOFA assessments. The median ICU length of stay was 1.91 days, and the postoperative hospital stay was 20.64 days. Overall, 79.56% of the patients (218 out of 274) survived to discharge (Table 1).”*

*“SOFA scores on the day of operation were similar between groups, whereas SOFA scores on postoperative day 7 (3 vs. 4, p = 0.007) and ΔSOFA values (−2 vs. 0, p < 0.001) differed. Organ dysfunction categories (improvement, stability, progression) also differed significantly between groups (p = 0.001). Other variables, including sex, comorbidities (e.g., hypertension, diabetes), lactate levels, and CRRT use, did not differ significantly. ICU and postoperative hospital stays were similar, but survival to discharge was higher in the post-RRS group (85.27% vs. 74.48%, p = 0.039) (Table 1).”*

Discussion section (Page 17)

*“Beyond improvements in timeliness, patients in the post-RRS period also demonstrated more favorable patterns of organ dysfunction. SOFA scores on the day of operation were similar between groups, but postoperative day 7 SOFA scores were lower in the RRS group, and the reduction in SOFA from baseline was greater, indicating that progression of organ dysfunction was mitigated after RRS activation. Consistent with this trend, fewer patients required vasopressor or ventilator support one week postoperatively, suggesting attenuation of physiologic deterioration. Although preoperative vasopressor and ventilator use was higher in the post-RRS group, the subsequent increase in postoperative organ support was not greater than that observed in the pre-RRS period, suggesting that RRS involvement may have helped stabilize patients before surgery. Collectively, these findings indicate that the benefits observed during the RRS period were not limited to earlier interventions but extended to improved postoperative physiologic trajectories and decreased organ support requirements. Further comparison of patients who presented during outside coverage hours showed substantially worse baseline severity, longer delays to operative management, and greater postoperative organ support needs, resulting in lower survival compared with patients evaluated during coverage hours.”*

Question 8: Unexplained Contradictory Results

Baseline data show that the postRRS group had higher rates of:

Preoperative vasopressor use (36.43% vs. 22.07%, p = 0.013);

Preoperative mechanical ventilation (20.93% vs. 6.90%, p = 0.001);

(These indicators suggest more severe preoperative illness.) However, there were no significant differences in postoperative vasopressor or mechanical ventilation rates between the two groups. The authors do not interpret "whether RRS reduces postoperative organ support needs by stabilizing preoperative condition," resulting in fragmented result interpretation .

Recommendation:

1. Calculate "the difference in organ support needs (preoperative vs. postoperative)" for each group (e.g., "postoperative vasopressor use rate minus preoperative rate");

2. Compare these differences between the preRRS and postRRS groups to clarify whether RRS mitigates the progression of illness (e.g., a smaller increase in postoperative organ support needs in the postRRS group would indicate RRS’s stabilizing effect) .

: We reviewed this comment carefully. As the reviewer suggested, we examined changes in organ support requirements from the preoperative to postoperative period in both groups. The results showed that although the post-RRS group had substantially higher preoperative severity, the degree of postoperative increase in vasopressor and mechanical ventilation use was not greater than that observed in the pre-RRS group.

Specifically,

• Vasopressor use increased by 27.6% in the pre-RRS group (22.07% → 49.66%),

 whereas the increase was 23.3% in the post-RRS group (36.43% → 59.69%).

• Mechanical ventilation increased by 40.7% in the pre-RRS group (6.90% → 47.59%),

 compared with 39.5% in the post-RRS group (20.93% → 60.47%).

These findings indicate that despite markedly worse baseline physiological derangement, the post-RRS group did not demonstrate a disproportionately greater escalation in postoperative organ support requirements. This pattern supports the interpretation that RRS activation may have stabilized patients preoperatively and mitigated further deterioration. This interpretation is consistent with the postoperative SOFA trajectories already reported in the manuscript. Although SOFA scores on the day of surgery were similar between groups, patients in the post-RRS period showed lower postoperative day 7 SOFA scores and greater reductions from baseline, suggesting attenuation of organ dysfunction progression after RRS implementation. This stabilizing effect is now highlighted in the revised Discussion.

The Revised text added to the manuscript (Material and method section, Page 17):

*“Beyond improvements in timeliness, patients in the post-RRS period also demonstrated more favorable patterns of organ dysfunction … Consistent with this trend, fewer patients required vasopressor or ventilator support one week postoperatively, suggesting attenuation of physiologic deterioration. Although preoperative vasopressor and ventilator use was higher in the post-RRS group, the subsequent increase in postoperative organ support was not greater than that observed in the pre-RRS period, suggesting that RRS involvement may have helped stabilize patients before surgery. Collectively, … Further comparison of patients who presented during outside coverage hours showed substantially worse baseline severity, longer delays to operative management, and greater postoperative organ support needs, resulting in lower survival compared with patients evaluated during coverage hours.”*

Question 9: Spelling Errors

1. "climinating variables sequentially" (Statistical Analysis section):

Error: "climinating" (extra "c");

Correction: "eliminating" (meaning "to remove variables stepwise") .

2. "intation of antibiotic administration" (Baseline Characteristics section):

Error: "intation" (incorrect term; "intation" refers to "endotracheal intubation," which is unrelated to antibiotic administration);

Correction: "initiation" (meaning "the start of antibiotic administration") .

3. "APACHI II" (Forest Plot variable label):

Error: Missing "E" (abbreviation error);

Correction: "APACHE II" (full name: Acute Physiology and Chronic Health Evaluation II, a standard severityofillness score) .

: We carefully re-examined the manuscript regarding the spelling issues noted by the reviewer.

For number 1 (“eliminating variables”) and number 2 (“initiation of antibiotic administration”), the manuscript already contains the corrected terms as suggested, and we could not identify the misspellings “climinating” or “intation” in our submitted version. These may have resulted from a file conversion or display issue during the review process. Nevertheless, we confirmed again that the correct terms (“eliminating” and “initiation”) are consistently used throughout the manuscript. For number 3, There was an inadvertent typographical error (“APACHI II”) in the earlier version of the forest plot. This has been fully corrected to “APACHE II” in the updated figure, which also reflects the revised model including lactic acid.

Question 10: Punctuation and Formatting Errors

1. Duplicate pvalue Labeling:

Original text (Baseline Characteristics section): "the time from diagnosis of acute abdomen to ICU admission (5.83 vs. 8.77 h, p < 0.001), \(p<0.001\) ), surgery (4.57 vs.6.48 h, \(p=0.036\) )"

Error: Duplicate pvalue label ("p < 0.001") for ICU admission time; missing space between "vs." and "6.48";

Correction: "the time from diagnosis of acute abdomen to ICU admission (5.83 vs. 8.77 h, p < 0.001), surgery (4.57 vs. 6.48 h, p = 0.036)" .

2. Missing Subject:

Original text (Baseline Characteristics section): "Vasopressor use and mechanical ventilation were higher in the postRRS group preoperatively (36.43% vs.22.07%, \(p=0.013\) and 20.93% vs.6.90%, \(p=0.001\) , respectively)"

Errors: Missing subject ("were higher" incorrectly refers to "vasopressor use/mechanical ventilation" instead of their "rates"); misspelling ("prcoperatively" → "preoperatively"); missing spaces around "vs.";

Correction: "The rates of vasopressor use and mechanical ventilation were higher in the postRRS group preoperatively (36.43% vs. 22.07%, p = 0.013 and 20.93% vs. 6.90%, p = 0.001, respectively)" .

3. Disorganized Table Formatting:

In Table 1, "Preoperative Fluid Administration (mL)" row:

Inconsistent number formatting: "1300.00" (postRRS group) lacks a thousands separator (should be "1,300.00");

Cell content overflow: Values (e.g., "300.00 (300.00–1,000.00)") exceed cell width, reducing readability.

Recommendations:

Standardize number formatting (add thousands separators for numbers ≥1,000);

Adjust column widths to ensure values fit within cells without overflow .

: We reviewed the issues noted regarding punctuation and formatting.

For numbers 1 and 2, the errors described (duplicate p-value labeling, misspellings, and missing spaces) were not present in our submitted manuscript. We suspect that these may have arisen from document conversion or display during the review process. We rechecked the revised manuscript, and the phrasing already appears in the corrected form as suggested by the reviewer. For number 3, we agree with the reviewer’s observation. The values in the “Preoperative Fluid Administration” row exceeded the cell width and impaired readability due to the mL scale. To improve clarity and consistency, we converted fluid administration units from mL to L and reformatted the table accordingly. The updated version is now easier to interpret without overflow or inconsistent number formatting.
